# Perioperative Chemotherapy in Gastroesophageal Cancer. A Retrospective Monocenter Evaluation of 42 Cases

**DOI:** 10.1371/journal.pone.0122974

**Published:** 2015-04-09

**Authors:** Ann-Christin E. Brehler, Wolfgang Hartmann, Stefanie Wiebe, Andrea Kerkhoff, Christoph Schliemann, Daniel Palmes, Norbert Senninger, Frank Lenze, Hansjoerg Ullerich, Wolfgang E. Berdel, Torsten Kessler

**Affiliations:** 1 Department of Medicine, Hematology and Oncology, University of Muenster, Muenster, Germany; 2 Gerhard Domagk Institute of Pathology, University of Muenster, Muenster, Germany; 3 Department of General and Visceral Surgery, University of Muenster, Muenster, Germany; 4 Department of Medicine, Gastroenterology, University of Muenster, Muenster, Germany; University of Algarve, PORTUGAL

## Abstract

**Background:**

Perioperative chemotherapy increases the overall and progression-free survival of patients suffering from resectable adenocarcinomas of the lower esophagus, gastroesophageal junction and stomach (GEC). Comparing different chemotherapy regimens platin-based protocols with 5-fluorouracil (5-FU)/calcium folinate (CF) or oral fluoropyrimidines were favorable in terms of efficacy and side-effects. However, there is no consensus which regimen is the most efficacious.

**Methods:**

42 consecutive patients with resectable GEC (UICC II and III) were treated with 3 pre- and postoperative chemotherapy cycles each consisting of epirubicin, oxaliplatin and capecitabine (EOX). We analyzed the overall survival, progression-free survival and toxicity retrospectively in comparison to published data.

**Results:**

The median overall survival in our cohort was 29 months and the progression-free survival was 17 months. The most frequent grade 3 and 4 toxicities during preoperative chemotherapy were diarrhea (16.7%), leukocytopenia (9.5%) and nausea (9.5%); overall 38.1% of our patients suffered from grade 3 or 4 toxicity. Surgery was carried out in 83% of our patients, 69% of those achieved R0 resection.

**Conclusion:**

Comparing our data with the results of previously published randomized trials EOX is at least non-inferior with regard to overall survival, progression-free survival and toxicity. In conclusion, EOX is an appropriate perioperative therapy for patients with resectable GEC.

## Introduction

Gastroesophageal adenocarcinomas of the lower esophagus, gastroesophageal junction and stomach (GEC) belong to the most common malignancies. The incidence of gastroesophageal junction and lower esophagus adenocarcinoma increases whereas the incidence of stomach cancer decreases [1,2]. Stomach cancer is still the fourth most common malignancy worldwide responsible for 738,000 deaths annually [3].

In early stages endoscopic mucosal resection, endoscopic submucosal dissection or minimal invasive surgery can be a curative therapy, but at the time of diagnosis most patients suffer from advanced or metastatic disease. Surgery is the favored treatment also for locally advanced cancer [4], however most patients have a relapse. 5-year-survival rates are about 10% for esophageal and about 21% for gastric cancer [5]. In advanced cancer neoadjuvant chemotherapy was added to reduce the tumor bulk and to eradicate micrometastasis [6]. In a meta-analysis neoadjuvant chemotherapy had a 2-year absolute survival benefit of 7% [7]. The limitation of this approach could be an insufficient eradication of micrometastasis, thus adjuvant chemotherapy was added [8]. The advantage of perioperative chemotherapy was documented in large scale randomized trials comparing different chemotherapy protocols [8]. The absolute 5-year-survival rate benefit was in the range of 10–15% with a hazard ratio of 0.6–0.8, regardless of the perioperative approach. Therefore different perioperative chemotherapy regimens are still used [8].

The MAGIC study by Cunningham et al. compared the effect of surgery alone with perioperative epirubicin, cisplatin and 5-FU/CF (ECF) chemotherapy in 503 patients suffering from resectable GEC [9]. Perioperative chemotherapy with ECF resulted in cancer regression and a significant extended progression-free and overall survival [9]. This approach was confirmed by another trial [10].

The REAL-2 study compared the efficacy of different perioperative chemotherapy protocols (ECF, ECX (epirubicin, cisplatin, capecitabine), EOF (epirubicin, oxaliplatin, 5-FU/CF), EOX (epirubicin, oxaliplatin, capecitabine)) in overall 1002 patients suffering from locally advanced (inoperable) or metastatic gastroesophageal cancer [11]. The longest survival time was observed for EOX treated patients. Overall, the authors concluded that EOX was as effective as ECF in patients with previously untreated GEC. Further clinical trials confirmed the high efficacy of the EOX protocol [12].

On the basis of these published studies we started to treat patients suffering from histologically proven resectable GEC in the perioperative setting routinely with a modified EOX protocol. Here we report a retrospective analysis of all our patients treated between January 2008 and September 2013 with regard to treatment efficacy and safety.

## Methods

### Patients

42 consecutive patients suffering from resectable gastroesophageal adenocarcinoma and fit for chemotherapy and radical surgery as determined by an interdisciplinary tumor board were recruited for EOX treatment. The pretreatment tumor staging comprised upper GI-tract endoscopy, radial endoscopic ultrasonography, and abdomen and thorax computed tomography according to the ESMO guidelines for esophageal and gastric cancer [13] [14]. Written informed consent was obligatory.

Main contraindications for EOX therapy were ECOG status ≥ 2 (Eastern Cooperative Oncology Group performance status), instable cardiac disease, hemodynamic relevant arrhythmia, renal impairment according to the drug product label (creatinine clearance <30ml/min) and inadequate cellular blood counts.

After confirmation by the Ethic Comitee at *Aerztekammer Westfalen-Lippe* (AEKWL) Germany, patient records were anonymized and de-identified prior to analysis.

### Treatment

The treatment protocol consisted of 3 preoperative and 3 postoperative EOX cycles each in intervals of 21 days. Each cycle consisted of epirubicin (50 mg/m^2^) by short infusion over 30 minutes followed by oxaliplatin (130 mg/m^2^) infused over 120 minutes on day 1. Capecitabine was given orally from day 1 to day 14 bidaily at a dose of 1250mg/m^2^ (2500 mg/m^2^/d). Infusions were administered by an intravenous port. Routinely dexamethasone, granisetron, clemastin and fosaprepitant were given for antiemetic prophylaxis, loperamide for diarrhea, and thrice daily 10% urea topically to prevent hand-and-foot-syndrome.

Clinical assessment was conducted by an experienced oncologist prior to chemotherapy. A complete cellular blood count, serum electrolytes, and liver and kidney function were determined. Relevant peripheral cytopenia resulted in dose adjustment; the capecitabine dose was reduced if severe diarrhea occurred or replaced by 5-FU/CF in an equivalent dose if patients were suffering from clinical relevant dysphagia.

Chemotherapy associated intolerance symptoms were documented according to the National Cancer Institute Common Terminology Criteria for Adverse Events (NCI CTCAE), v3.0. Continuation of the therapy regimen was individually discussed with patients dependent on subjective and objective tolerability.

Therapy efficacy was evaluated by upper GI-tract endoscopy, radial endoscopic ultrasonography, CT or PET-CT preoperatively. The radiologic response was calculated according to RECIST criteria [15]. In case of tumor regression the surgical intervention was conducted 3–6 weeks after the third chemotherapy cycle. Chemotherapy continuation or premature surgery was individually discussed with patients in the case of stable disease. Disease progression resulted in chemotherapy cessation and accelerated surgical intervention.

The procedure was determined according to the tumor site by the surgeon. Staging was supplemented by histopathological evaluation of the surgical specimen, the histopathological regression due to chemotherapy was documented according to the Baldus et al. classification system (Grade 1–4, 2004) [16]. The downstaging rate was calculated comparing the initial clinical with the pathological tumor and nodal state.

Postoperative chemotherapy was initiated 6 to 12 weeks after surgery with 3 cycles of EOX in 3 weekly intervals.

Follow-up care frequency was 3 months during the first follow-up year, 6 months during the second year, then once a year. Clinical inspection, upper GI-tract endoscopy, CT-scan according to tumor site and abdominal ultrasound were performed routinely, CA 72–4 and CEA were analyzed in patients with elevated values at the time of diagnosis. Our database was closed at 26th of March 2014.

### Statistical Analysis

The primary objective of this evaluation was the calculation of progression-free (PFS) and overall survival (OS) of patients treated by pre- and postoperative EOX chemotherapy in comparison with data from the literature. Overall survival (OS) is the time between the initial diagnosis and the date of death from any cause or the last day of follow-up. Progression-free survival (PFS) is the time from diagnosis to the date of relapse, disease progression, or the last day of follow-up. Kaplan-Meier curves were calculated with IBM SPSS Statistics 22.

Further outreads of this analysis were the operability after chemotherapy with the intent of a complete resection (R0), treatment toxicity, pathological response rate, down-staging by chemotherapy, and Baldus regression status.

Prognostic factors (gender, tumor size, histological tumor grading, histopathological nodal state, and tolerability of chemotherapy) were analyzed by the use of Kaplan-Meier log-rank test and Cox univariate regression analysis. The values of statistically significant factors were evaluated in a multivariate stepwise forward Cox regression model.

## Results

### Characteristics of patients

The characteristics of our 42 patients are summarized in Table 1. 40 patients suffered from cancer of stage group UICC II and UICC III. 1 patient was included despite diagnosis of a resectable solitary liver metastasis (UICC IV) and 1 patient with UICC stage Ib. The majority of our patients were male. The median age was 61.5 years. None had a previous chemotherapy or concurrent malignancy. All patients had a good performance status according to the ECOG scale (26 patients ECOG 0 and 16 patients ECOG 1).

**Table 1:** Pretherapeutical patient’s and tumor characteristics, and tumor localization.

****Variable****	****Value****
Age at diagnosis: range	35.7–82.1 years
Median (years)	61.55
Gender	
Male (n)	36 (85.7%)
Female (n)	6 (14.3%)
Performance status (ECOG scale)	
0	26
1	16
Tumor localization	
Esophagus (n)	3
Junction AEG 1–3 (n)	29
AEG 1	17
AEG 2	10
AEG 3	2
Stomach (n)	10
Clinical T stage	
T 1 (n)	0
T 2 (n)	8
T 3 (n)	30
T 4 (n)	4
Clinical UICC stage	
UICC I	1 (2.4%)
UICC II	11 (26.2%)
UICC III	29 (69.0%)
UICC IV	1 (2.4%)
Maximum tumor diameter (n = 38)	
0.0–3.9cm	10 (26.3%)
4.0–7.9cm	24 (63.2%)
8.0–11.9cm	4 (10.5%)
Grading	
G1	3 (7.1%)
G2	11 (26.2%)
G3	28 (66.7%)

### Treatment

#### Preoperative chemotherapy

The median time between diagnosis and chemotherapy was 32 days (standard deviation (SD) 16.6 days) with a minimum of 14 and a maximum of 105 days.

The complete preoperative regimen (3 chemotherapy cycles) was administrated to 31 patients; 7 patients had 2 and 4 patients 1 cycle. Reasons for cessation of preoperative chemotherapy were: toxic effects in 10 patients (severe intestinal side effects (n = 3), severe hematotoxicity (n = 2), severe stomatitis (n = 1), hand-foot syndrome (n = 1), cardiac ischemia (n = 1), acute renal failure (n = 1) and laryngopharyngeal spasm (n = 1)).

#### Surgical intervention

2 patients died during the preoperative phase (1 progress related death and 1 death after completion of 3 preoperative cycles), in 5 patients surgery was cancelled (in 3 due to progress, 2 patients refused surgery). 35 (83.3%) of the initial 42 patients had a surgical procedure (with the purpose of gastrectomy, transhiatal extended cardia resection or distal esophagectomy): R0 resection was documented in 24/35 (68.6%), R1 resection in 6 (17.1%), R2 resection in 2 patients (5.7%), of these 1 patient underwent a palliative R2-gastrectomy with lymphadenectomia and the other a tumor bulk reduction with reconstruction of the intestinal passage. In 2 patients the resection was stated as RX (5.7%). In 1 patient surgery was determined after explorative laparotomy. The median time between diagnosis and surgery was 121 days (SD 24.6 days), and between preoperative chemotherapy and surgery 32 days (SD 14.7 days).

#### Postoperative chemotherapy

Postoperative chemotherapy was restarted in 14 patients (40%) in median 47.5 (SD 23.2 days) after the procedure. A second postoperative cycle could be administered in 11 and a third in 10 patients.

Chemotherapy restart was precluded by intolerable previous toxicity or treatment inefficacy in 9 patients and patients request or postoperative complications in 12 patients. The subsequent treatment was determined after discussion in our tumor review board on an individual base.

#### Chemotherapy adverse effects

The EOX associated adverse effects during the pre- and postsurgical phases are summarized in Table 2. Grade 3 or 4 toxicity was observed in 16 patients (38.1%) during preoperative chemotherapy with need for inpatient admission in 16 individuals. Postoperatively grade 3 or 4 toxicity was observed in 4 patients (28.6%). A reduction of chemotherapeutic agents dose was documented in 31 therapy cycles applied to 12 patients.

**Table 2:** Chemotherapy related adverse effects during preoperative and postoperative treatment phase.

****Adverse effect****	****Preoperative n (%)****			****Postoperative n****		
	*Grade 0*	*Grade 1 or 2*	*Grade 3 or 4*	*Grade 0*	*Grade 1 or 2*	*Grade 3 or 4*
**Hematologic**						
Anemia	30 (71.4%)	12 (28.6%)	0	9	5	
Thrombocytopenia	36 (85.7%)	5 (11.9%)	1 (2.4%)	12	2	
Leukocytopenia	19 (45.2%)	19 (45.2%)	4 (9.5%)	7	5	2
**Non haematologic**						
Nausea	10 (23.8%)	28 (66.7%)	4 (9.5%)	0	14	0
Vomiting	30 (71.4%)	12 (28.6%)	0	6	8	0
Diarrhea	25 (59.5%)	10 (23.8%)	7 (16.7%)	5	8	1
Hand-foot syndrome	40 (95.2%)		2 (4.8%)	14	0	0
Neurologic Effects	35 (83.3%)	7 (16.7%)	0	11	1	2
Thrombosis	39 (92.9%)	0	3 (7.1%)	13	0	1
Stomatitis	40 (95.2%)	1 (2.4%)	1 (2.4%)	14	0	0
Cardiac Ischemia	39 (92.9%)	0	3 (7.1%)	14	0	0

### Efficacy

Preoperative chemotherapy efficacy and response rates are summarized in Table 3. By radiological evaluation 38 out of 40 patients (95%) with at least 1 cycle had a partial response (47.5%) or stable disease (47.5%) prior to surgery. Comparing the pathological stage with the baseline clinical stage a TNM downstaging was found in 18/35 patients (51.4%). A major response to chemotherapy defined as histopathological regression grade 3+4 (less than 10% residual tumor) was found in 10 patients (30.3%), of these 1 had a complete regression; grade 2 regression was documented in 8 patients (24.2%), and grade 1 regression in 15 (45.5%).

**Table 3:** Response rates to preoperative EOX chemotherapy.

	****Variable****	****N (%)****
**Radiologic response after preoperative EOX**	Partial response	19/42 (45.2)
	Stable disease	19/42 (45.2)
	Progressive disease	2/42 (4.8)
	Not done	2/42 (4.8)
**Downstaging after surgery**	Yes	18/35 (51.4)
	No	17/35 (48.6)
**Histopathological regression Baldus**	Grade 1 (>50% residual tumor)	15/33 (45.5)
	Grade 2 (10–50% residual tumor)	8/33 (24.2)
	Grade 3 (<10% residual tumor)	9/33 (27.3)
	Grade 4 (complete regression)	1/33 (3.0)

The median follow-up period was 19 months (range 1–49 months). 1 patient was lost to follow-up. 24 patients (57.1%) had disease progression, in median after 10 months (range 1–30 months). During the evaluation period 20 patients died (48%): 16 cancer related, 1 during postsurgical phase, 1 prior to surgery with unknown cause of death and 2 during follow-up (1 cardiac disease, 1 unknown cause).

**Fig 1:**
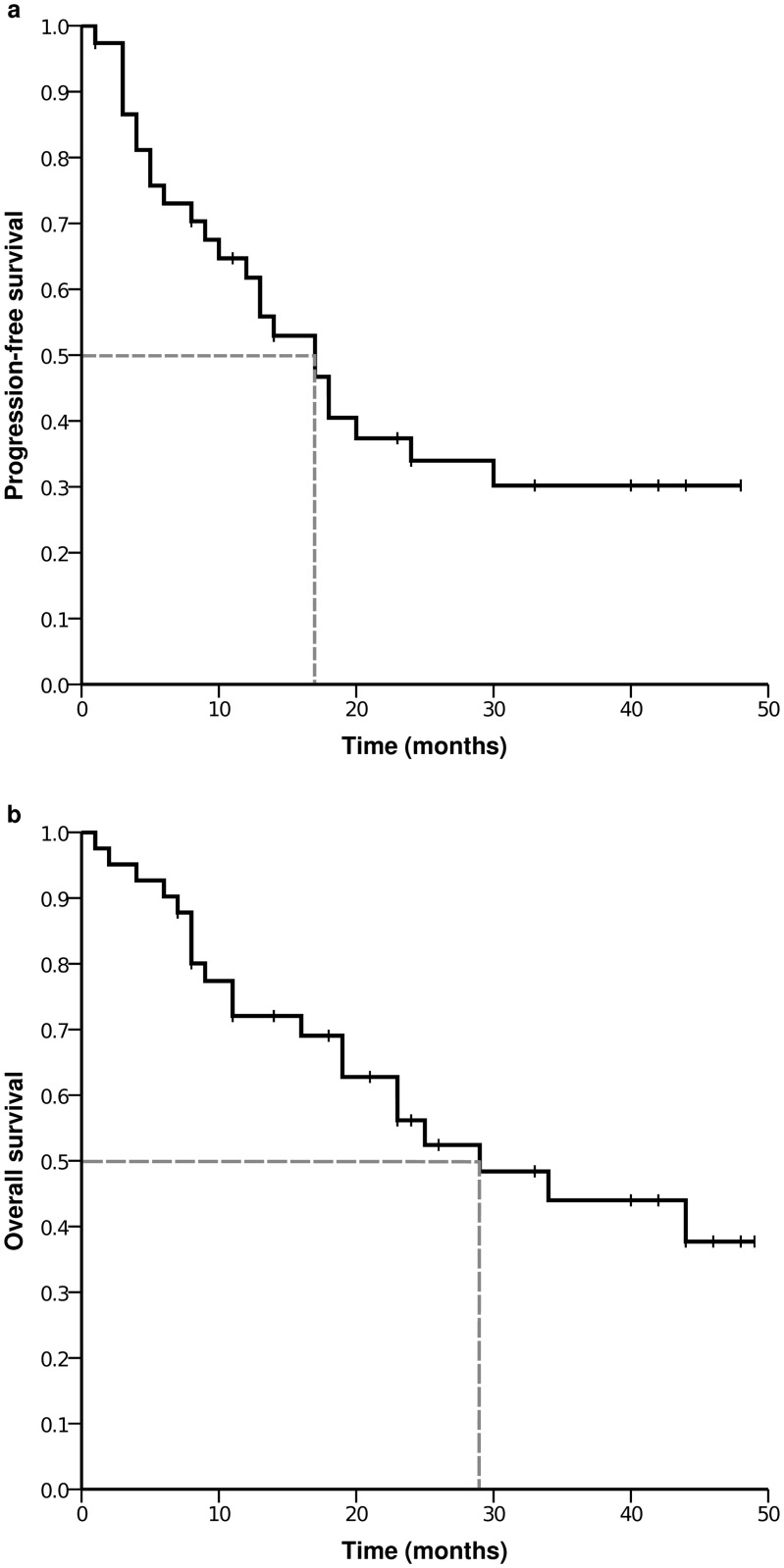
Kaplan-Meier plots of progression-free survival (a) and overall survival (b).

**Fig 2:**
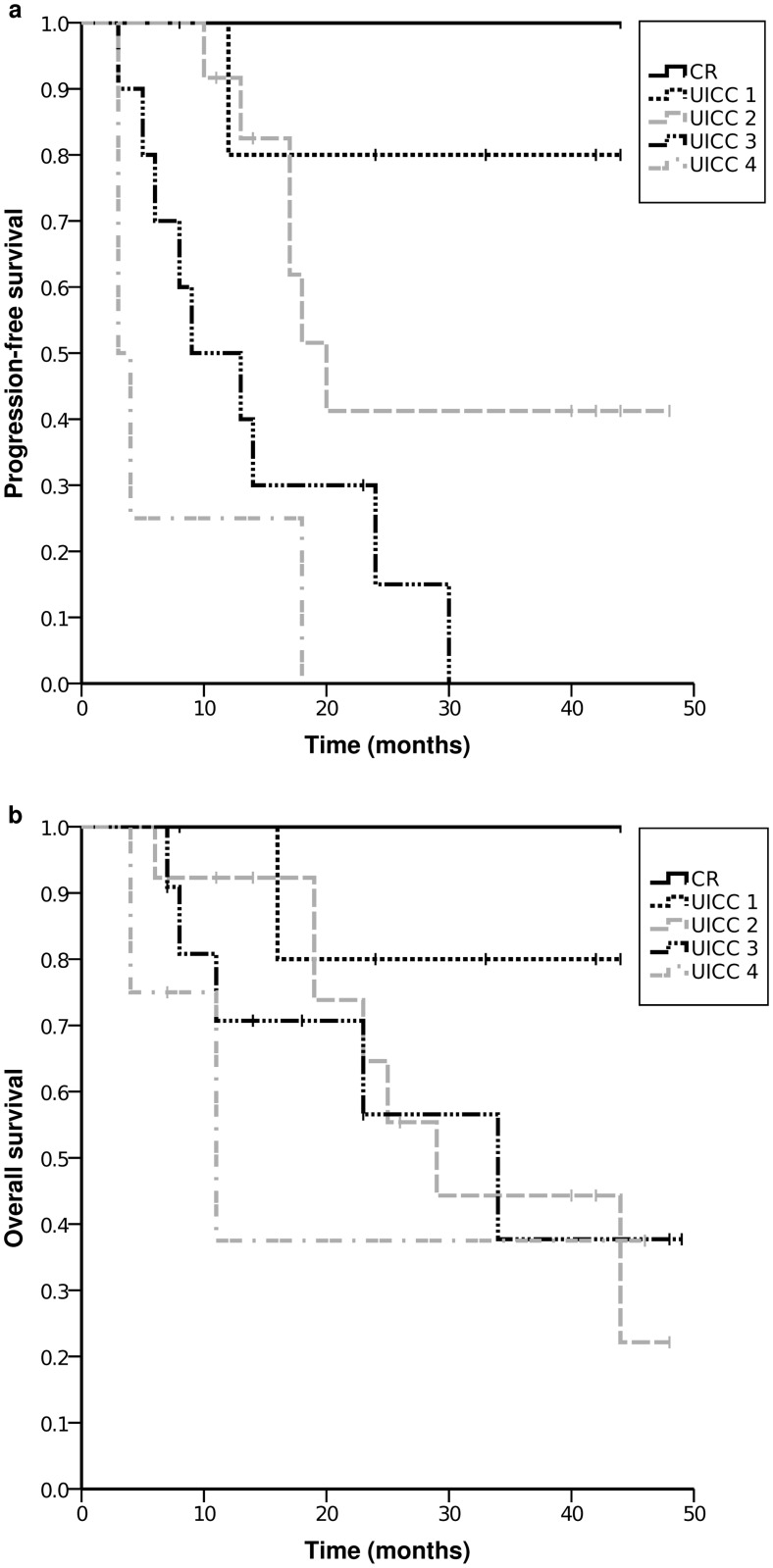
Kaplan-Meier plots of PFS (a) and OS (b) according to postsurgical UICC stages.

For the complete patient group the median PFS was 17 months (95%CI 11.6–22.4) and the median OS was 29 months (95%CI 13.3–44.7). Kaplan-Meier curves of PFS and OS are given in Fig 1. For patients with surgical intervention the median PFS was 18 months (95%CI 11.9–24.1, mean 24.8) and the median OS 34 months (95%CI 11.7–56.3, mean 33.2). In Fig 2 the Kaplan-Meier curves of PFS and OS are given according to the postsurgical UICC stages. The PFS was significantly higher for UICC stage 0, I and II each compared with the PFS of patients with the higher stages. The PFS for patients was not significantly different between UICC stage III and IV. Comparing the OS of patients according to UICC stages differences were not significant.

In the univariate log-rank test a negative histopathological nodal status ypN (*p* = 0.012), a high number of preoperative (*p* = 0.042) and at least one postoperative chemotherapy cycle(s) (*p* = 0.011), R0 resection (*p* = 0.013), downstaging by chemotherapy (*p* = 0.006), and complete or nearly complete regression of the tumor after preoperative chemotherapy (Baldus regression grade 3 and 4) (*p* = 0.015) were significantly related to a longer PFS. Significant positive prognostic factors for OS were 3 preoperative (*p* = 0.031) and at least 1 postoperative chemotherapy cycle (*p* = 0.042), chemotherapy hematotoxicity (*p =* 0.028) and the ability of surgical intervention (*p*<0.001).

In the multivariate Cox regression analysis only the lymph node status at the time of surgical intervention had a significant impact on PFS, the statistical significance of all other factors were lost in the multivariate analysis.

## Discussion and Conclusions

Here we report our experience with perioperative EOX chemotherapy in a treatment regimen for patients suffering from principally resectable advanced gastroesophageal adenocarcinomas. Compared to data in the literature we find that this approach was highly efficacious, and toxicity was well tolerable. Considering those patients of our study having undergone surgery the median PFS was 18 months and the median OS 34 months. As a result of the small number of patients the OS did not correlate with postsurgical UICC stages as expected, but the PFS was longer in UICC stage 0, I and II patients.

The efficacy of different chemotherapy protocols was evaluated in the REAL-2 study in palliative patients suffering from inoperable or metastatic GEC [11]. EOX was favorable due to the toxicity profile, the differences between the median survival times were not statistically significant (9.9 months for ECF, 9.9 months for ECX, 9.3 months for EOF, and 11.2 months for EOX). In a NICE statement from 28th July 2010 capecitabine has been recommended in combination with platinum-based chemotherapy protocols as a first-line treatment of inoperable advanced gastric cancer [17]. A clear recommendation for the perioperative situation does not exist so far.

The MAGIC study had demonstrated a better outcome of perioperative ECF chemotherapy in comparison to surgery alone for patients suffering from principally resectable GEC with regard to PFS (hazard ratio 0.66) and OS (hazard ratio 0.75) [9]. The five-year survival rate after surgery alone was 23% but 36.3% for patients with additional perioperative chemotherapy. We replaced cisplatin by oxaliplatin and 5-FU/CF by capecitabine on the base of the REAL-2 study and modified the chemotherapy protocol; we administered capecitabine in a higher dose (2500 mg/m^2^) over a shorter period (14 days). Patient characteristics (age, gender, tumor stage) in the MAGIC study and our patient collective were comparable. The difference in the median follow-up period (49 months in the MAGIC study for patients in the perioperative chemotherapy group, and 19 months in our patients collective) represents a limitation for comparability. According to the presented MAGIC trial data it could be estimated that the median PFS was about 18 months and the median OS about 25 months for the patients in the perioperative chemotherapy group.

Our data give some evidence that the number of chemotherapy cycles is related to longer OS. Patients treated with a minimum of one post-operative chemotherapy cycle had a trend for longer OS with a mean of 38.1 months versus 28.3 month (*p* = 0.042) for patients without postoperative chemotherapy.

In the literature the toxicity data for capecitabine were not inferior to 5-FU/CF although there was evidence of some differences in adverse event profiles [17]. In a clinical trial on 224 patients suffering from resectable GEC patients were treated with 2 or 3 preoperative and 3 or 4 cycles postoperative cycles consisting of cisplatin and 5-FU/CF [10]. Grade 3 or 4 toxicity was reported in 41 patients (38%) due to preoperative chemotherapy, in our patient collective this were 16 out of 42 patients (38.1%). Hematotoxicity was relatively mild in our patients with grade 3 and 4 leukocytopenia in 9.5%, thrombocytopenia in 2.4%, severe anemia was not observed. Grade 3 or 4 hematotoxicity during postoperative chemotherapy was only observed in 2/14 patients, both suffered from leukocytopenia. We found that the incidence of grade 3 and 4 toxicity due to the combination of 3 chemotherapeutic agents was comparable with a regimen consisting of cisplatin and 5-FU/CF [10].

To date all chemotherapy protocols for patients suffering from advanced GEC are based on 5-FU/CF or oral fluoropyrimdine in combination with either cisplatin or oxaliplatin. The addition of epirubicin as third agent has been shown to be more effective associated with an acceptable increase of toxicity. With all the limitations of a retrospective analysis our data support the hypothesis that it is safe to modify the original MAGIC regimen by replacement of cisplatin with oxaliplatin and the 21-day infusion of 5-FU/CF with capecitabine in the perioperative setting without losing efficacy.

Recently, the efficacy of other treatment protocols for potentially curable GEC has been published. The addition of docetaxel instead of epirubicin is another approach in preoperative and perioperative regimens [18–20]. The efficacy of neoadjuvant chemoradiotherapy in the preoperative setting has been approved, representing an alternative approach to chemotherapy alone [21].

The debate on the optimal chemotherapy regimen in this setting is not closed, although it is unlikely that there will be a head to head comparison of different protocols. The introduction of new substances in treatment protocols for patients suffering from resectable GEC such as trastuzumab which already has been approved for the metastatic situation in Her2/NEU positive GEC is currently under evaluation. New protocols combining classical chemotherapy and biological agents have been promising in early studies, but often failed in randomized phase III trials. One example is the combination of EOX and panitumumab which resulted in an unacceptable level of toxicity and was less efficacious compared with EOX [22,23]. However, the EOX regimen is one standard regimen in first line therapy for metastatic GEC and we think our data supports its use also in the perioperative setting.

Finally, there is still an unmet need for further prospective trials to define multimodality treatment protocols with better efficacy and tolerability.
